# Involvement of Proinflammatory Arachidonic Acid (ARA) Derivatives in Crohn’s Disease (CD) and Ulcerative Colitis (UC)

**DOI:** 10.3390/jcm11071861

**Published:** 2022-03-27

**Authors:** Justyna Kikut, Małgorzata Mokrzycka, Arleta Drozd, Urszula Grzybowska-Chlebowczyk, Maciej Ziętek, Małgorzata Szczuko

**Affiliations:** 1 Department of Human Nutrition and Metabolomics, Pomeranian Medical University in Szczecin, W. Broniewskiego 24, 71-460 Szczecin, Poland; justyna.kikut@pum.edu.pl (J.K.); arleta.drozd@pum.edu.pl (A.D.); 2 Department of Pediatrics, Hemato-Oncology and Pediatric Gastroenterology, Independent Public Clinical Hospital No.1 of Pomeranian Medical University in Szczecin, Unii Lubelskiej 1, 71-252 Szczecin, Poland; mokrzycka.mal@gmail.com; 3 Department of Pediatrics, Faculty of Medical Sciences in Katowice, Medical University of Silesia in Katowice, Medyków 16, 40-752 Katowice, Poland; uchlebowczyk@sum.edu.pl; 4 Department of Perinatology, Obstetrics and Gynecology Pomeranian Medical University in Szczecin, Siedlecka 2, 72-010 Police, Poland; maciej.zietek@pum.edu.pl

**Keywords:** Crohn’s disease, ulcerative colitis, inflammatory bowel disease, adolescents, eicosanoids, 15-HETE, 9HODE, lipoxins

## Abstract

Recently, an increase in the incidence of inflammatory bowel disease (IBD) has been observed, especially among children and adolescents. Currently, few studies focus on the differentiation of inflammation in IBD subunits, i.e., Crohn’s Disease (CD) and Ulcerative Colitis (UC). The aim of this study was to compare the concentrations of proinflammatory mediators of arachidonic acid (ARA) and linoleic acid (LA) in patients with CD (*n* = 34) and UC (*n* = 30), in order to identify differences in inflammation in both diseases and within the same entity, according to disease activity. Sixty-four adolescents with a mean age of 13.76 ± 2.69 and 14.15 ± 3.31, for CD and UC, respectively, were enrolled in the study. Biochemical analysis of ARA and LA derivatives was performed using a liquid chromatography. A trend was observed in the concentration of 15S-HETE (hydroxyeicosatetraenoic acids) in CD relative to UC. The active phase of both diseases showed a higher 15S-HETE concentration in active CD relative to active UC. Comparing patients with CD with active and inactive disease showed a trend of increased levels of thromboxane B2, leukotriene *B4* and 9S-HODE (hydroxyoctadecadienoic acid) in the active versus the inactive disease. We also observed statistically significantly higher levels of 12S-HETE in inactive CD relative to active CD. In the UC group, on the other hand, statistically significantly higher levels of prostaglandin E2 and 16RS-HETE were observed in active UC relative to inactive UC. Moreover, significantly higher concentrations of LTX A4 5S, 6R were observed in inactive UC relative to the active phase. In conclusion, the present study indicated the activity of the 15-LOX pathway in CD. Further studies involving lipid mediators in patients with IBD may contribute to the development of new therapies for the treatment of IBD. The identification of differences in the course of inflammation may help to target therapy in CD and UC, and perhaps allow the introduction of an additional diagnostic marker between the two main IBD subtypes.

## 1. Introduction

The term inflammatory bowel disease (IBD) refers to two disease entities, i.e., Crohn’s disease (CD) and Ulcerative Colitis (UC). IBD is characterized by chronic inflammation in the gastrointestinal mucosa [1]. However, inflammation in CD may involve any part of the entire gastrointestinal tract [2], whereas inflammation in UC involves the colon and rectum [3]. The etiology of the disease has not been precisely defined; it is suggested that it may be associated with genetic predisposition, environmental influence, abnormal immune response and coexistence of dysbiosis [4]. Currently, the incidence of IBD is increasing worldwide. Although in Western countries the incidence is gradually stabilizing, in newly industrialized countries it is still increasing [5,6]. Unfortunately, the increase in IBD incidence is also observed in children and adolescents. It is estimated that as many as 25% of patients have their first symptoms before the age of 18 [7]. The disease in children is characterized by a more severe and aggressive course. In addition, delayed growth and puberty is observed in children [8,9]. 

At the moment, the treatment of IBD is based on induction and maintenance therapy. The most commonly used pharmacotherapies include aminosalicylates, steroids and immunosuppressive drugs, as well as monoclonal antibody therapy. Recently, however, more and more studies are based on the search for non-invasive markers that could be useful for the diagnosis and monitoring of the disease [10]. At the moment, still only a few markers of inflammation in blood or serum have been validated in relation to IBD. The most commonly available and routinely used are c-reactive protein (CRP) and the erythrocyte sedimentation rate (ESR). Unfortunately, the markers have different half-lives and also vary in sensitivity [11]. Currently, one of the better inflammatory markers used for monitoring IBD is fecal calprotectin [12,13]. Finally, the blood markers CRP and fecal calprotectin are considered reliable and useful in monitoring the disease [14].

Despite the many therapeutic strategies used in IBD, unfortunately, some patients do not respond positively to the implemented treatment which leads to serious consequences. Moreover, although pharmacology has a positive effect, it is often associated with impaired functioning of the immune response. In addition, some patients do not respond to treatment at all or lose response to the drug over time; hence, there is a constant need to search for new therapeutic strategies in IBD. Therefore, as noted by Camba-Gomez et al., new therapeutic options based on a reduction in inflammation through the use of, e.g., pro-resolving mediators should be developed [15,16]. So far, it has been established that a higher long-term intake of omega 3 polyunsaturated fatty acids (PUFAs, n-3) is associated with a lower risk of UC in adult women [17]. Moreover, UC patients have been shown to have higher ARA and lower LA concentrations in the colonic mucosa compared to controls [18]. When compared to mucosa without inflammation, the fatty acid ratio indicates higher bioavailability of ARA [18]. It has also been observed that with increasing pro-resolution mediators, including lipoxins concentrations, and decreasing leukotrienes, the patient enters a remission phase of the disease. In the opposite situation, the disease persists or the patient does not respond to treatment [19].

**Figure 1:**
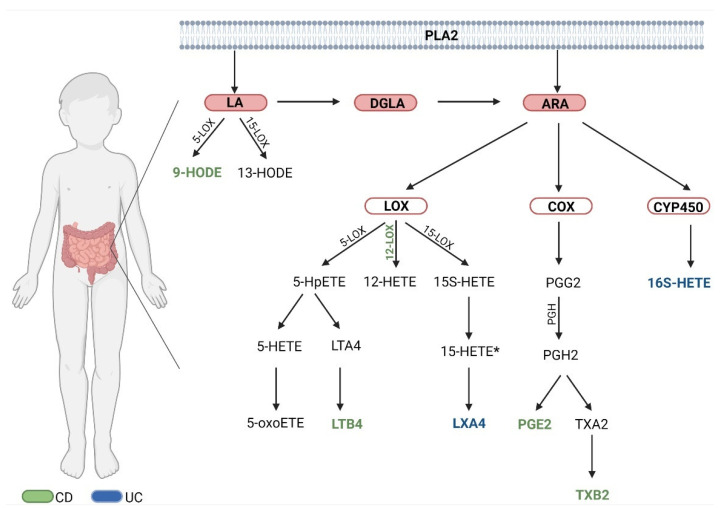
Synthesis of HETE and HODE acids analyzed in the study. PLA2—Phospholipase A2; LA—Linoleic acid; DGLA—Dihomo-γ-linolenic acid; ARA—Arachidonic acid; Green font—meaning in CD; Blue font—meaning in UC; *—significant difference between CD and UC (total); Created with BioRender.com (accessed on 1 January 2022).

Lipids are responsible for cell membrane integrity and fluidity, intercellular signaling, and eicosanoid production itself. The disturbance of these functions significantly affects the expression and maintenance of inflammation and thus may lead to the development of IBD [20]. Moreover, it has been suggested that inflammation in IBD may be a consequence of the disruption of the mechanisms regulating the inflammatory response [21]. Eicosanoids are formed by three enzymes, i.e., cyclooxygenases (COX-1, COX-2), lipoxygenases (LOX-5, -8, -12, -15) and cytochromes P450 (CYP450), from three major acids, i.e., linoleic acid (LA), dihomo-γ-linolenic acid (DGLA), arachidonic acid (ARA), as well as eicosapentaenoic acid (EPA) and docosahexaenoic acid (DHA). Arachidonic acid is the main precursor for the synthesis of eicosanoids [22]. Subsequently, pro-inflammatory lipid mediators such as hydroxyeicosatetraenoic acid (HETE), prostaglandins, thromboxanes, leukotrienes, and lipoxins are formed from ARA and hydroxyoctadecadienoic acid (HODE) is formed from LA [23,24]. A diagram showing the eicosanoids analyzed in this study is shown below (Figure 1). Some of the more important cited ARA pathways in IBD are COX-1, COX-2, and 5- and 12-lipoxygenase (12-LOX) [25]. Other authors emphasize that inflammation in IBD is characterized by higher levels of eicosanoids derived from ARA metabolism, such as prostaglandin E, prostaglandin 2, and leukotrienes B4 [26]. In the early stage of inflammation, the expression of the proinflammatory enzymes 5-lipoxygenase (5-LOX) and COX-2 increases leading to the synthesis of leukotriene B4 (LTB4) and leukotriene C4 (LTC4) and prostaglandin E2 (PGE2) and prostaglandin D2 (PGD2) [27]. Lipoxins provide a signal to inhibit inflammation and are formed from ARA with the involvement of 5- and 15-lipoxygenase [28]. Lipoxin A4 (LXA4) also inhibits neutrophil chemotaxis and epithelial adhesion [29]. Insufficient lipoxin production in UC has also been associated with dysregulation of 5- and 15-lipoxygenase activity [28]. It has been suggested that the inhibition of enzymes such as 5-LOX and COX-2, that exert proinflammatory effects, may be helpful in developing treatments for inflammatory diseases [27]. PGE2 is associated with inflammation promotion [30]. PGE2 via LTB4 enhances tissue damage, which in turn can be blocked by LXA4 [31]. It has also been shown that PGE2 can act as an irritant to the intestinal lumen, relax the colonic muscles and contribute to diarrhea [32]. It has been suggested that IBD may be caused by the inadequate expression of pro- and anti-inflammatory molecules that are associated with the immune system [33].

The aim of this study was to compare the concentrations of the proinflammatory mediators ARA and LA in patients with CD and UC in order to identify differences in the course of inflammation in both diseases and within the same entity, according to disease activity. To date, there are limited reports on mediator concentrations in the active disease form of both entities (CD and UC).

## 2. Results

### 2.1. Characteristics of the Study Group

A total of 64 patients with confirmed Crohn’s disease or Ulcerative Colitis were eligible for the study. The CD group included 34 subjects, while the UC group included 30 subjects. The study participants were hospitalized in two university departments of gastroenterology. IBD was diagnosed by endoscopic examination using high quality medical endoscope by Olympus (China) with histopathological evaluation (tissue samples taken during endoscopic examination). In addition, the patients underwent ultrasonography and, in the case of CD, enteroclysis or enterography with computed tomography (CT) imaging. The inclusion criteria were diagnosed CD or UC and participants aged between 7 and 18 years. Exclusion criteria were parenteral nutrition implemented, the use of specialized elimination diets (such as gluten-free diet, Crohn’s disease exclusion diet (CDED)). During recruitment, six patients refused to participate in the study, representing approximately 10% of the study population. The characteristics of the population are shown in Table 1.

### 2.2. Division of the Study Group

In the CD patients’ group, 10 patients were in remission and 24 patients were in the active phase of the disease. Among the UC patients, 6 patients were in remission and 24 patients presented with the active phase of the disease. The Pediatric Crohn’s Disease Activity Index (PCDAI) or the Pediatric Ulcerative Colitis Activity Index (PUCAI) was used to assess disease activity. The patients with CD and UC received the following groups of medications: aminosalicylates, corticosteroids, immunomodulatory drugs, and biologic therapy. Referring to supplementation therapy in the CD and UC group, patients were mostly treated with vitamin D, probiotics, calcium, and iron. Additionally, the CD group patients were supplemented with B vitamins and the UC group patients with potassium. Medication and supplementation data are presented in Table 2.

Slightly higher concentrations of TXB2, prostaglandin E2, leukotriene B4, 13S-HODE, 9S-HODE, and 5-HETE were observed in the CD group relative to UC (however, the differences were not statistically significant). A trend in 15S-HETE concentration was observed in CD relative to UC (*p* = 0.09) (Table 3).

In the next step, the mean levels of the lipid mediators were compared between patients with active and inactive disease. Referring to the CD group, there was a trend of increasing levels of TXB2 (*p* = 0.079), LTB4 (*p* = 0.083) and 9S-HODE (*p* = 0.087) in the active versus the inactive disease (however, these differences were not statistically significant). In addition, 12S-HETE (*p* = 0.033) was observed to be significantly higher in the inactive versus the active disease (Table 2). In the UC group, on the other hand, significantly higher levels of PGE2 (*p* = 0.009) and 16RS-HETE (*p* = 0.038) were observed in the active versus the inactive disease. In addition, significantly higher concentrations of LTX A4 5S, 6R (*p* = 0.034) were observed in inactive UC relative to the active phase (Table 2). Comparing the active phase of both diseases, a higher 15S-HETE concentration was shown in active CD relative to active UC (*p* = 0.051) (Table 2). In the inactive phase of the diseases (remission), no differences in the concentration of the studied mediators were observed between the studied entities. (Table 4).

## 3. Discussion

There is a lack of knowledge on the involvement of proinflammatory mediators in inflammatory bowel disease distinguishing the two disease entities (CD and UC) and two phases (active and remission). The few studies that have been conducted so far usually involved small groups of patients. Recruitment to the study group itself varied and the treatment of patients was usually not uniform. Moreover, the parameters were not studied in groups of children but only in adults, often without regard to gender. All these differences significantly influenced the interpretation of the results obtained by the authors of these studies. Our study concerns the largest group of patients examined so far with both disease entities and in the two phases of the diseases. There is no doubt that proinflammatory mediators derived from ARA are involved in the course of the disease [34,35,36]. However, such a broad profile of derivatives of both disease entities has not been compared to each other so far. At the same time, no studies on LA derivatives (HODE) have been found.

### 3.1. Comparison of Both Diseases (CD vs. UC)

Analysis of the active phase of both diseases (CD and UC) showed a trend in 15S-HETE levels in active CD relative to active UC. This may indicate a greater involvement of the 15-LOX pathway in the course of CD. As in other studies, our study confirmed increased 5-LOX activity in CD relative to UC. Higher levels of 12S-HETE were also observed in active UC relative to active CD, suggesting that the 12-HETE pathway is more involved in UC than in CD. Additionally, in the present study, we observed an increased proportion of 9-,13-HODE in both active CD and remission CD relative to both phases of UC, which in turn may suggest the relevance of the LA pathway in the course of CD to a greater extent than in UC.

15-HETE, as the predominant mediator, was observed in biopsies from UC patients relative to healthy individuals [37], which can be explained by the fact that 15S-HETE is associated with effects on cell proliferation and inflammation development [38]. Another study has also shown that the main eicosanoid identified in inflamed colon tissue was 15-HETE [39]. In another study, increased 5-LOX pathway activity and increased leukotriene B4 synthesis were observed in patients with active IBD compared to controls [40]. Additionally, higher levels of leukotriene B4 have been demonstrated in the colonic mucosa of IBD patients compared to controls [36]. In IBD, a significant infiltration of neutrophils is observed which probably affects the higher activation of LTB4 [20]. LTB4, as a proinflammatory product of eicosanoid metabolism, activates the influx and activation of leukocytes and neutrophils at the site of inflammation [41]. Studies have shown that LTB4, together with lipoxygenase products, are among the major metabolites identified in the mucosa of IBD patients and are at much higher concentrations compared to healthy subjects. It seems that the increased synthesis of LTB4 may be responsible for the inflammatory response in IBD in general [42]. A study by Jupp et al. demonstrated higher 5-LOX activity and higher LTB4 levels in biopsy material from patients with active disease relative to controls [34]. On the other hand, in a study by Ikehata et al., 5-LOX activity was higher in CD than in UC [35]. Such a relationship was also observed in the present study. In opposition to our study, a higher activity of eicosanoids from the COX pathway was observed in UC compared to CD, which may be a result of the selection of the group of patients studied (smaller than our group) [34]. However, in our study, the concentration of one of the COX pathway mediators, i.e., PGE2, was also slightly higher in the active phase of UC relative to CD, although in both disease entities it increases dramatically in the active phase of the disease relative to remission. Lauritsen et al. studied ARA metabolite levels in rectal dialysates and similarly observed higher PGE2 levels in UC patients relative to CD. In contrast to our results, TXB2 and LTB4 levels were higher in UC patients than CD patients [43]. In contrast, Baumeister et al. suggested that PGE2 may be one of the major eicosanoids in less severe inflammatory bowel disease [44]. Higher concentrations of the vast majority of inflammatory mediators were also observed in patients with CD compared to UC. This suggests that the involvement of ARA and LA mediators is more intense in CD than in UC. With respect to the results obtained from healthy adults, we can observe significantly higher concentrations of PGE2, 12S-HETE, 15S-HETE, and 5-HETE in both study groups [45].

New to the study is an attempt to compare the remission phase of both diseases. Most studies focus on comparing phases of active disease. Analysis of the remission phase of both diseases (CD and UC) showed higher levels of PGE2 and 12S-HETE in the remission phase of CD relative to UC and significantly higher levels of LTX A4 5S, 6R in UC relative to CD. Additionally, we observed higher levels of 5-oxoETE in UC remission relative to CD, which may indicate the involvement of this eicosanoid in the induction of UC remission. Interestingly, 5-HETE levels do not change significantly between the phases of both diseases as well as between the diseases themselves. This may indicate that 5-HETE will differentiate the diseases in question to a lesser extent. Additionally, 5-HETE is rapidly oxidized to 5-oxoETE, and oxidative stress enhances its production. Furthermore, 5-oxoETE is also a more potent eicosanoid than 5-HETE and additionally, has been shown to act synergistically with other lipid mediators. The authors suggest that 5-oxoETE may enhance the movement of neutrophils and eosinophils in chronic inflammation [46]. Other authors have associated higher levels of 5-oxoETE in the colonic mucosa of patients with irritable bowel syndrome with predominant constipation [47]. Another study also indicates that the production of lipid mediators changes significantly with the progression of active inflammatory bowel disease [48]. This may also indicate that the limitations of the study include the fact that it did not assume that the study group was divided according to the medications taken and did not take into account changes due to medications taken in remission. Further research should also include pharmacological partitioning, degree of disease activity, and etiological and genetic differences. Ultimately, the experience of selecting the appropriate dose for age and stage of disease should be tested. The research which is analyzed in the discussion is summarized in Table 5.

**Table 5:** Summary table of the research presented in the discussion.

References	IBD/UC/CD	Participants	Sample	Results
**IBD**				
Jupp et al., 2007 [34]	IBD	Study group (*n* = 23) active disease *n* = 17 (CD *n* = 7, UC *n* = 9, nonspecific IBD *n* = 1) men (*n* = 9) women (*n* = 8) average age 47.2 inactive disease (*n* = 6) men (*n* = 3) women (*n* = 3) average age 52.0 Control group (*n* = 9)	Colonic biopsies	higher 5-LOX activity and higher LTB4 levels in biopsy material from patients with active disease relative to controls changes in eicosanoid concentrations more markedly observed in the UC group relative to controls
Ikehata et al., 1994 [35]	IBD	Study group (*n* = 17) untreated active UC (*n* = 11) men (*n* = 7) women (*n* = 4) average age 39.3 untreated active CD (*n* = 6) men (*n* = 4) women (*n* = 2) average age 23.7 Control group (*n* = 5)	Colonic mucosa	5-LOX activity was higher in CD than in UC, especially in CD compared to mucosa of UC patients without inflammation
Sharon et al., 1984 [36]	IBD	Study group (*n* = 11 ) men (*n* = 5) women (*n* = 4) age 17–61 Control group (*n* = 9)	Colonic mucosa	higher levels of TXB2, LTB4 and 5-HETE in the colonic mucosa of IBD patients
Lauritsen et al., 1988 [43]	IBD untreated patients	Study group (*n* = 37) active UC (*n* = 20) men (*n* = 10) women (*n* = 10) average age 29.0 (19–69) active CD (*n* = 10) men (*n* = 3) women (*n* = 7) average age 30.0 (20–34) Clostridium difficile colitis (*n* = 7) men (*n* = 1) women (*n* = 6) average age 36.0 (22–68) Control group (*n* = 10)	Dialysis of the rectum	higher concentration of PGE2, LTB4, TXB2 in UC relative to CD
Baumeister et al., 1996 [44]	IBD	Study group (*n* = 7) Control group (*n* = 10)	Colonic mucosa	increased PGE2 production in IBD relative to controls PGE2 may be one of the major eicosanoids in less severe IBD
Shannon et al., 1993 [49]	IBD Active disease	Study group (*n* = 8) active UC (*n* = 4) active CD (*n* = 4) men (*n* = 5), women (*n* = 3) age 26–49 Control group (*n* = 8)	Colonic mucosa samples from inflamed and non-inflamed tissue	increased 12-lipoxygenase activity in inflamed regions of the colon in IBD compared to controls (12-HETE increased in IBD)
**ACTIVE UC**				
Zijlstra et al., 1992 [37]	UC	Study group of active UC (*n* = 11) men (*n* = 8) women (*n* = 3) age 21–64 Control group (*n* = 13)	Colonic tissue	the main eicosanoid identified in inflamed colon tissue was 15-HETE 12-HETE, PGF2, PGE2, TXB2 were present in much lower concentrations than 15-HETE
Zijlstra et al., 1992 [39]	UC	Study group of active UC (*n* = 11) men (*n* = 8) women (*n* = 3) age 21–64 Control group (*n* = 13) *	Colonic tissue	15-HETE as the predominant mediator was observed in biopsies from UC patients relative to healthy individuals
Masoodi et al., 2013 [50]	UC	Study group of active UC (*n* = 54) men (*n* = 26) women (*n* = 28) average age 44.4 ± 1.8 Control group (*n* = 42)	Colonic mucosa biopsy	levels of 5-, 11-, 12-, and 15-HETE were found in the study group relative to healthy controls higher levels of PGE2 and TXB2 were observed in the mucosa of patients compared to healthy controls 5-, 11-, 12-, 15-HEPE mediators were indeterminate in biopsy material from these patients
Zijlstra et al., 1991 [51]	UC	man with mild proctocolitis in histology (*n* = 1) age 35	Mucus from morning stool	15-HETE was identified in the highest amount in the patient’s mucus, followed by LTB4, TXB2 and PGE2 which were also identified in lower amounts
**ACTIVE AND INACTIVE UC**				
Hamabata et al., 2018 [48]	DSS-induced Colitis in mice	-	Colon tissue	production of lipid mediators changes significantly with the progression of active inflammatory bowel disease
Gewirtz et al., 2002 [52]	DSS-induced Colitis in mice	-	-	Oral administration of LXA(4) analog (10g per day) resulted in slowed weight loss and reduced mortality, and resolution of inflammation
Vong et al., 2012 [53]	UC	Study group (*n* = 24) active UC (*n* = 8) men (*n* = 5) women (*n* = 3) average age 47 ± 11 inactive UC (*n* = 16) men (*n* = 9) women (*n* = 7) average age 44 ± 13 Control group (*n* = 20)	Colonic mucosa biopsies	LXA(4) higher in biopsies from patients in remission only upregulation of AnxA1 protein expression in inactive group
Fiorucci et al., 2004 [54]	TNBS-induced Colitis in mice (CD model)	-	Plasma and colonic mucosa	12-HETE-PE, which can be formed from 12-LOX is removed in the acute phase of peritonitis and reappears in the resolution of acute inflammation ZK-192 (oral pharmacokinetics) and related 3-oxa-ATL analog may have a therapeutic function in CD
Diab et al., 2019 [55]	UC	Study group (*n* = 20) active, newly diagnosed untreated UC (*n* = 15) men (*n* = 9) women (*n* = 6) average age 37.0 (14–69) inactive UC (*n* = 5) men (*n* = 5) average age 46.0 (41–70) Control group (*n* = 10)	Colon biopsies	higher levels of 15S-HETE in remission patients relative to healthy patients
**CD**				
Pochard et al., 2016 [56]	CD	Study group (*n* = 6) active disease (n=6) men (*n* = 3) women (*n* = 3) age 19–74 Control group (*n* = 6) Sprague Dawley rats	Cultures of human and adult rat enteric glial cells (EGC)	15-HETE production in EGC CD patients was reduced compared to controls 15-HETE inhibition in rats increased intestinal epithelial barrier permeability

UC—Ulcerative Colitis, CD—Crohn’s Disease, IBD—Inflammatory Bowel Disease, DSS—dextran sodium sulfate, TNBS—Trinitrobenzene Sulfonic Acid. * patients untreated for at least one month.

### 3.2. Comparison of Both Phases in CD

Analyzing the active phase of CD with the remission phase of CD, we captured a trend in the increase in LTB4 and TXB2, similarly to 9S-HODE. Although there was no statistical significance in relation to PGE2 levels, this can be explained by the fact that the standard deviation in the group of patients with active disease was quite high and the difference in mediator levels between the active phase and remission was significant. We also observed a statistically significantly higher concentration of 12S-HETE mediator in remission CD relative to active CD. This may suggest activation of the 12S-HETE pathway in the resolution of inflammation and the participation of the pathway in inducing remission in CD, which confirms the results of previous tests. A novelty in the study is determining 9S-HODE levels in the pediatric group. Although the lack of statistical differences between the active phase and inactive disease coincides with studies in adults.

Analyzing the literature data, intracellularly 12-HETE is associated with oxidative stress, while extracellular activity of this eicosanoid affects signaling pathways modulating inflammatory activity. The involvement of the 12-HETE pathway in liver damage or diabetes, among others, has also been demonstrated [40]. In IBD, higher levels of 12-HETE were identified in the active phase of the disease in several studies [36,49]. According to Kulkarni at al., elevated 12S-HETE levels in the remission phase may be an early prognostic marker [40]. On the other hand, in another study 12-HETE-PE, which can be formed from 12-LOX is removed in the acute phase of peritonitis and reappears in the resolution of acute inflammation [57], which may also explain the increase in 12-HETE activity during the remission phase in our study.

### 3.3. Comparison of Both Phases in UC

When we analyzed the active phase of UC with the remission phase of UC, we observed a statistically significantly higher concentration of PGE2 in the active phase of UC relative to remission. Moreover, a significantly higher concentration of LTXA4 was observed in UC remission relative to the active phase of the disease, which was not observed in the CD group. In addition, statistically significantly higher levels were also associated with 16RS-HETE, which was higher in the active phase of UC compared to remission. However, the phase of the disease that the patients were in was not stated. A novelty in this study is the 16RS-HETE assay, which has not been extensively studied. All three ARA pathways, including cyclooxygenase, lipoxygenase and CYP450, are involved in the active phase of UC. However, the contribution of PGE2 and 16RS-HETE is associated with the proinflammatory effect, while LTX A4 5S, 6R is associated with the silent state. Perhaps in the active phase of UC, lipoxin analogues could be used to induce remission.

Currently, studies show a positive effect of lipoxin analogues in controlling inflammation and they may have therapeutic functions in diseases characterized by mucosal inflammation [52,58]. Moreover, synthetic analogues of LXA4 have higher activity and are more stable. Similarly, a study by Vong et al. showed higher levels of LXA4 in mucosal biopsies from patients in remission [53]. Other studies in mice and cell cultures have linked the effect of LXA4 to the resolution of inflammation [52,54]. In a study by Bednar et al., it was observed that 16R-HETE is produced by human multinucleated leukocytes and in rabbit studies it inhibited the progression of thromboembolic stroke making. The authors suggest that it could be used for therapeutic purposes in various diseases that develop on the basis of inflammation [59]. Other studied mediators such as LTB4, 9-, 13-HODE, 5-, 12-, 15-HETE were slightly decreased in remission relative to active disease. In a study by Masoodi et al. using mucosal biopsies from adult UC patients, elevated levels of 5-, 11-, 12-, and 15-HETE were found in the study group relative to healthy controls. Additionally, significantly higher levels of PGE2 and TXB2 were observed in the mucosa of patients compared to healthy controls. Interestingly, 5-, 11-, 12-, and 15-HEPE mediators were indeterminate in biopsy material from these patients [50]. Mucosal biopsies from UC patients showed significantly higher levels of 15S-HETE in remission patients relative to healthy patients [55]. A study by Pochard et al. found that CD patients have a lower production of 15-HETE relative to controls, which affects the increased intestinal permeability [56]. In another study, LTB4 and 15-HETE were identified in the stool mucus of a UC patient at significantly higher levels than PGE2 and thromboxane B2 [51].

## 4. Materials and Methods

The study was conducted after obtaining approval from the Bioethics Committee of the Pomeranian Medical University (KB-0012/131/18 dated 26 November 2018). Patients’ participation in the study has been voluntary. Written consents to participate in the study were obtained from the legal guardians of adolescent patients and they were also informed about the possibility of withdrawal from participation. Patients over 16 years of age also gave such a written consent.

### 4.1. Anthropometric Measurements

To assess the differences in the nutritional status of the patients, anthropometric measurements such as body weight (±0.1 kg) and height (±0.5 cm) were collected, and for their accurate determination, a medical scale (Radwag WPT 60/150 OW, Poland) with a height meter was used. Anthropometric measurements were taken during the visit, with the use of centile grids (for body weight and BMI) OLA and OLAF, recommended by the “Child Health Center” Institute (Warsaw, Poland) [60].

### 4.2. Sample Collection

Venous blood was collected into tubes on EDTA bed. The blood was centrifuged (3500 rpm for 10 min), separated for plasma and morphotic components into Eppendorf tubes (500 µL) and stored in a freezer at −80 degrees C until the analysis of the mediators. Biochemical analysis was performed using a liquid chromatography (HPLC) apparatus.

### 4.3. Extraction of Eicosanoids

Arachidonic acid and linoleic acid derivatives were extracted from the plasma using an RP-18 SPE column (Agilent Technologies, UK). Standards of ARA and LA derivatives: Thromboxane B2 (TXB2), Prostaglandin E2 (PGE2), Leukotriene B4 (LTB4), 13S-HODE (13S-hydroxy-9Z,11E-octadecadienoic acid), 9S-HODE (9S-hydroxy-10E, 12Z-octadecadienoic acid), 15S-HETE (15S-hydroxy-5Z,8Z,11Z,13E-eicosatetraenoic acid), 12S-HETE (12S-hydroxy-5Z,8Z,10E,14Z-eicosatetraenoic acid), 5-oxoETE (5-Oxo-eicosatetraenoic acid), 5-HETE (5-hydroxyeicosatetraenoic acid), 5(S),6(R)-lipoxin-A4 (5S,6R,15S-trihydroxy-7E,9E,11Z,13E-eicosatetraenoic acid), 5(S),6(R)15(R)-lipoxin-A4, (5(S),6(R),15(R)-trihydroxy 7E,9E,11Z,13E-eicosatetraenoic acid), 16RS-HETE Cayman Chemicals (Ann Arbor, MI, USA).

400 µL of serum, 50 µL of PGB (prostaglandin B) internal standard and 1 mL of chilled acetonitrile were added to an Eppendorf. The samples were then mixed on vortex for one minute and incubated for 20 min at −20 degrees C. After incubation, the samples were centrifuged for 15 min (14,000 rpm). Subsequently, the obtained supernatant was transferred into 7 mL tubes and 4.5 mL of 1 mM HCl was added. Then, 1 M HCl was added to obtain pH = 3. In the next step, the columns were conditioned with 100% acetonitrile and 20% acetonitrile in 1 mM HCl. The supernatant was then applied to the columns and washed twice with 3 mL of 20% acetonitrile in 1 mM HCl. The samples were eluted into Eppendorfs with a mixture of methanol and ethyl acetate (1:1). The samples were then analyzed on a liquid chromatography apparatus (Agilent Technologies 1260). The dedicated Agilent ChemStation software (Agilent Technologies, Cheadle, UK) was used to control and analyze the results. The method was described in more detail in our previous article [45].

### 4.4. Statistical Analysis

Statistical analysis was performed using Statistica 13.3 software (StatSoft, Cracow, Poland). Data were analyzed by the Shapiro–Wilk test for normality of distribution; all data showed normal distribution. Comparative analysis of diseases in the active phase was performed using the Student’s T-test. The analysis between the active and remission phase of the diseases and between the diseases in remission has been performed using the nonparametric Mann–Whitney U test. A value of *p* < 0.05 was taken as the level of statistical significance.

## 5. Conclusions

In conclusion, this study demonstrated the involvement of the AA and LA pathways in the course of both disease entities. Moreover, this study confirmed previous reports of 12S-HETE involvement in the active phase of CD. It can be concluded that the activity of the pathway was more intense in CD than in UC, although no significant differences were found in the course of inflammation in the active phase of both diseases (due to a significant deviation). However, remission concerned the activity of the 15-LOX pathway in CD. The decreases in TXB2, PGE2, LTB4 and 9-HODE activity may be important elements of therapy planning, as they may increase 12-LOX activity in CD. Decreased activity in PGE2, 16RS-HETE and activation of the pathway leading to LXA4 synthesis may be a point of intervention in UC. In contrast, 15-HETE may be a marker that differentiates the two entities in the active phase (Figure 1). It is worth adding that this study had its limitations, even though it includes one of the largest numbers of patients with IBD. More research is required on this topic with more participants in remission and with the comparison of the results to a healthy control group. Further research may help to target therapy in CD and UC, and possibly introduce an additional diagnostic marker between these two main IBD subtypes. It is extremely important to identify additional markers analyzed from the serum in order to avoid conducting invasive endoscopic studies.

## Figures and Tables

**Table 1:** Characteristics of patients with CD and UC.

Parameter	CD Avg ± SD *n* = 34	UC Avg ± SD *n* = 30	*p*-Value
Age (years)	13.76 ± 2.69	14.15 ± 3.31	0.70
Body mass (kg)	46.81 ± 18.07	53.02 ± 19.40	0.18
Height (m)	1.54 ± 0.19	1.60 ± 0.20	0.26
Disease duration (months)	23.38 ± 26.45	19.57 ± 30.22	0.25
BMI (kg/m^2^)	19.06 ± 4.29	20.06 ± 4.91	0.40
BMI percentiles	43.09 ± 35.21	47.03 ± 37.74	0.71
Body mass percentiles	39.36 ± 34.76	46.40 ± 37.50	0.46
PCDAI	15.84 ± 16.08	-	-
PUCAI	-	30.00 ± 23.36	-
Fecal calprotectin active disease (µg/g)	2606.68 ± 2504.64	2230.07 ± 2113.7	0.66
Fecal calprotectin (µg/g)	2040.45 ± 2269.53	2096.77 ± 2110.49	0.94

PCDAI—Pediatric Crohn’s Disease Activity Index; PUCAI—Pediatric Ulcerative Colitis Activity Index; avg—average; SD—standard deviation, *p*-value < 0.05.

**Table 2:** Characteristics of the medications and supplements taken in the study groups.

	CD (*n* = 34)	UC (*n* = 30)
**Pharmacology**		
Aminosalicylates	23	24
Glucocorticosteroids	9	1
Immunomodulatory drugs	15	3
Biologic drugs	9	5
**Supplementation**		
Vitamin D	26	19
Probiotics	16	12
Calcium	11	7
Iron	5	6
B vitamins	8	-
Potassium	-	4

**Table 3:** Characteristics of selected lipid mediators in CD and UC groups.

Lipid Mediators (µg/mL)	CD Avg ± SD *n* = 34	UC Avg ± SD *n* = 30	*p*-Value
TXB2	0.090 ± 0.08	0.079 ± 0.07	0.614
PGE2	10.533 ± 30.46	9.633 ± 28.62	0.909
LTX A4 5S, 6R	0.099 ± 0.12	0.105 ± 0.14	0.863
LTX A4 5S, 6R, 15R	0.089 ± 0.11	0.087 ± 0.09	0.944
LTB4	0.102 ± 0.08	0.093 ± 0.07	0.678
16RS-HETE	0.540 ± 0.58	0.618 ± 0.42	0.567
13S-HODE	0.355 ± 0.40	0.274 ± 0.31	0.397
9S-HODE	0.428 ± 0.47	0.316 ± 0.33	0.308
15S-HETE	1.048 ± 0.79	0.758 ± 0.38	0.092
12S-HETE	3.404 ± 2.47	3.789 ± 3.89	0.649
5-oxo ETE	0.836 ± 0.72	0.852 ± 0.88	0.940
5-HETE	2.408 ± 1.37	2.244 ± 1.91	0.705

PGE2—Prostaglandin E2; LTB4—Leukotriene B4; CD—Crohn’s disease; UC—Ulcerative Colitis; avg—average; SD—standard deviation, *p*-value < 0.05.

**Table 4:** Characteristics of selected lipid mediators in CD and UC groups in patients with active disease and remission.

Mediators of the Inflammatory State (µg/mL)	Active CD Avg ± SD *n* = 24	Remission CD Avg ± SD *n* = 9	*p*-Value	Active UC Avg ± SD *n* = 24	Remission UC Avg ± SD *n* = 6	*p*-Value	*p*-Value CD vs. UC Active	*p*-Value CD vs. UC Remission
TXB2	0.106 ± 0.09	0.047 ± 0.06	0.079	0.082 ± 0.06	0.080 ± 0.08	0.575	0.321	0.875
PGE2	5.000 ± 15.93	1.808 ± 2.48	0.590	6.806 ± 23.19	0.470 ± 0.39	0.009	0.767	0.128
LTX A4 5S, 6R	0.098 ± 0.13	0.093 ± 0.12	0.815	0.082 ± 0.10	0.238 ± 0.25	0.034	0.638	0.156
LTX A4 5S, 6R, 15R	0.079 ± 0.12	0.102 ± 0.10	0.313	0.090 ± 0.09	0.073 ± 0.06	0.788	0.747	0.564
LTB4	0.115 ± 0.08	0.061 ± 0.08	0.083	0.097 ± 0.08	0.080 ± 0.05	0.643	0.465	0.318
16RS-HETE	0.562 ± 0.67	0.481 ± 0.28	0.673	0.694 ± 0.40	0.374 ± 0.35	0.038	0.443	0.958
13S-HODE	0.366 ± 0.34	0.327 ± 0.58	0.153	0.288 ± 0.32	0.254 ± 0.27	0.575	0.446	0.875
9S-HODE	0.445 ± 0.36	0.379 ± 0.74	0.087	0.341 ± 0.35	0.242 ± 0.29	0.643	0.339	0.713
15S-HETE	1.178 ± 0.87	0.844 ± 0.55	0.439	0.759 ± 0.41	0.742 ± 0.25	0.788	0.051	0.564
12S-HETE	2.741 ± 2.37	4.579 ± 2.16	0.033	3.919 ± 4.24	3.127 ± 2.17	0.942	0.264	0.104
5-oxo ETE	0.934 ± 0.82	0.616 ± 0.44	0.496	0.776 ± 0.71	1.376 ± 1.48	0.449	0.504	0.431
5-HETE	2.523 ± 1.52	2.198 ± 0.95	0.622	2.321 ± 2.11	2.069 ± 0.53	0.510	0.720	0.875

PGE2—Prostaglandin E2; LTB4—Leukotriene B4; LTX A4—lipoksyna A4; CD—Crohn’s disease; UC—Ulcerative Colitis;  avg—average; SD—standard deviation, *p*-value < 0.05.

## Data Availability

Not applicable.
